# 4,7-Dichloro­quinoline

**DOI:** 10.1107/S1600536812014924

**Published:** 2012-04-21

**Authors:** Amol A. Kulkarni, Christopher King, Ray J. Butcher, Joseph M. D. Fortunak

**Affiliations:** a College of Pharmacy, Howard University, 2300 4th Street, NW, Washington, DC 2059, USA; b Department of Chemistry, Howard University, 525 College Street, NW, Washington, DC 2059, USA

## Abstract

The two mol­ecules in the asymmetric unit of the title compound, C_9_H_5_Cl_2_N, are both essentially planar (r.m.s. deviations for all non-H atoms = 0.014 and 0.026 Å). There are no close C—H⋯Cl contacts.

## Related literature

4,7-dichloro­quinoline is a commonly used starting material for the synthesis of a variety of anti-malarial drugs, such as amodiquine {systematic name: 4-[(7-chloro­quinolin-4-yl)amino]-2-[(diethyl­amino)­meth­yl]phenol}, see: Dongre *et al.* (2007 ▶); O’Neill *et al.* (2003 ▶); Lawrence *et al.* (2008 ▶); Saha *et al.* (2009 ▶).
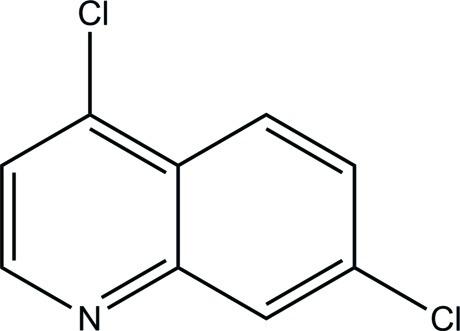


## Experimental

### 

#### Crystal data

C_9_H_5_Cl_2_N
*M* *_r_* = 198.04
Monoclinic, 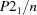

*a* = 18.2243 (17) Å
*b* = 3.8253 (5) Å
*c* = 23.622 (3) Å
β = 96.61 (1)°
*V* = 1635.8 (4) Å^3^
*Z* = 8
Cu *K*α radiation
μ = 6.59 mm^−1^
*T* = 123 K
0.35 × 0.23 × 0.16 mm

#### Data collection

Oxford Diffraction Xcalibur Ruby Gemini diffractometer
Absorption correction: multi-scan (*CrysAlis PRO*; Oxford Diffraction, 2007 ▶) *T*_min_ = 0.233, *T*_max_ = 1.000
5147 measured reflections
3188 independent reflections
2148 reflections with *I* > 2σ(*I*)
*R*_int_ = 0.090

#### Refinement

*R*[*F* ^2^ > 2σ(*F* ^2^)] = 0.096
*wR*(*F* ^2^) = 0.327
*S* = 1.08
3188 reflections
217 parameters
H-atom parameters constrained
Δρ_max_ = 0.68 e Å^−3^
Δρ_min_ = −0.49 e Å^−3^

### 

Data collection: *CrysAlis PRO* (Oxford Diffraction, 2007 ▶); cell refinement: *CrysAlis PRO*; data reduction: *CrysAlis PRO*; program(s) used to solve structure: *SHELXS97* (Sheldrick, 2008 ▶); program(s) used to refine structure: *SHELXL97* (Sheldrick, 2008 ▶); molecular graphics: *SHELXTL* (Sheldrick, 2008 ▶); software used to prepare material for publication: *SHELXTL*.

## Supplementary Material

Crystal structure: contains datablock(s) I, global. DOI: 10.1107/S1600536812014924/bt5833sup1.cif

Structure factors: contains datablock(s) I. DOI: 10.1107/S1600536812014924/bt5833Isup2.hkl

Supplementary material file. DOI: 10.1107/S1600536812014924/bt5833Isup3.cml

Additional supplementary materials:  crystallographic information; 3D view; checkCIF report

## Figures and Tables

**Fig. 1.**
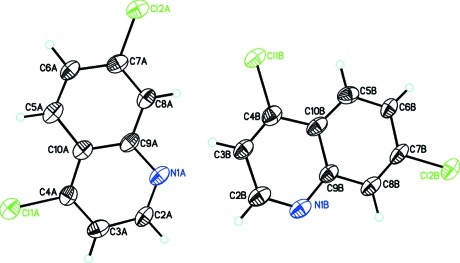
A view of the title compound, C9H5Cl2N, showing atom numbering scheme and the two molecules in the asymmetric unit.

## supplementary crystallographic information

### Comment

The crystal structure of 4,7-dichloroquinoline has not previously been reported. Recrystallization of 4,7-dichloroquinoline from hexane or similar hydrocarbon solvents removes low levels (1–4%) of 4,5-dichloroquinoline that are present from the manufacturing process. Impurities that arise from the presence of 4,5-dichloroquinoline in 4,7-DCQ are otherwise difficult to remove from the manufacturing process of commercial malaria drugs, including amodiaquine and piperaquine (Dongre *et al.*, 2007).

In view of the importance of this pharmaceutically active compound its crystal structure was determined. There are two molecules in the asymmetric unit (*Z*' = 2) and there are no close C—H···Cl contacts.

### Experimental

Hexanes (100 ml) were transferred to an Erlenmeyer flask and heated to a gentle reflux. 4,7-Dichloroquinoline (20 g, commercially available from Sigma-Aldrich) was slowly added to hexanes and the solution was maintained at 65 °C, resulting in a colorless solution. The solution was slowly cooled to room temperature and maintained at room temperature for 12 h. Long, colorless needles were observed to slowly crystallize from solution. The colorless needles obtained were isolated by filtration and dried to a constant weight, mp 83–84 °C; ^1^H-NMR (CDCl_3_) d 8.78 (d, J = 4.8 Hz, 1H), 8.15 (d, J = 9.2 Hz, 1H), 8.11 (d, J = 2.4 Hz, 1H), 7.59 (dd, J = 9.2, 2.4 Hz, 1H), 7.48 (d, J = 4.8 Hz, 1H).

### Refinement

H atoms were placed in geometrically idealized positions and constrained to ride on their parent atoms with a C—H distance of 0.95Å and *U*(H) = 1.2*U*_eq_(C).

### Crystal data

**Table:**

C_9_H_5_Cl_2_N	*F*(000) = 800
*M**_r_* = 198.04	*D*_x_ = 1.608 Mg m^−^^3^
Monoclinic, *P*2_1_/*n*	Cu *K*α radiation, λ = 1.54178 Å
Hall symbol: -P 2yn	Cell parameters from 1283 reflections
*a* = 18.2243 (17) Å	θ = 2.9–75.6°
*b* = 3.8253 (5) Å	µ = 6.59 mm^−^^1^
*c* = 23.622 (3) Å	*T* = 123 K
β = 96.61 (1)°	Prism, colorless
*V* = 1635.8 (4) Å^3^	0.35 × 0.23 × 0.16 mm
*Z* = 8	

### Data collection

**Table:**

Oxford Diffraction Xcalibur Ruby Gemini diffractometer	3188 independent reflections
Radiation source: Enhance (Cu) X-ray Source	2148 reflections with *I* > 2σ(*I*)
Graphite monochromator	*R*_int_ = 0.090
Detector resolution: 10.5081 pixels mm^-1^	θ_max_ = 75.8°, θ_min_ = 2.9°
ω scans	*h* = −22→16
Absorption correction: multi-scan (*CrysAlis PRO*; Oxford Diffraction, 2007)	*k* = −4→4
*T*_min_ = 0.233, *T*_max_ = 1.000	*l* = −23→29
5147 measured reflections	

### Refinement

**Table:**

Refinement on *F*^2^	Primary atom site location: structure-invariant direct methods
Least-squares matrix: full	Secondary atom site location: difference Fourier map
*R*[*F*^2^ > 2σ(*F*^2^)] = 0.096	Hydrogen site location: inferred from neighbouring sites
*wR*(*F*^2^) = 0.327	H-atom parameters constrained
*S* = 1.08	*w* = 1/[σ^2^(*F*_o_^2^) + (0.1577*P*)^2^ + 4.2615*P*] where *P* = (*F*_o_^2^ + 2*F*_c_^2^)/3
3188 reflections	(Δ/σ)_max_ < 0.001
217 parameters	Δρ_max_ = 0.68 e Å^−^^3^
0 restraints	Δρ_min_ = −0.49 e Å^−^^3^

### Special details

**Table:**

Geometry. All e.s.d.'s (except the e.s.d. in the dihedral angle between two l.s. planes) are estimated using the full covariance matrix. The cell e.s.d.'s are taken into account individually in the estimation of e.s.d.'s in distances, angles and torsion angles; correlations between e.s.d.'s in cell parameters are only used when they are defined by crystal symmetry. An approximate (isotropic) treatment of cell e.s.d.'s is used for estimating e.s.d.'s involving l.s. planes.
Refinement. Refinement of *F*^2^ against ALL reflections. The weighted *R*-factor *wR* and goodness of fit *S* are based on *F*^2^, conventional *R*-factors *R* are based on *F*, with *F* set to zero for negative *F*^2^. The threshold expression of *F*^2^ > σ(*F*^2^) is used only for calculating *R*-factors(gt) *etc*. and is not relevant to the choice of reflections for refinement. *R*-factors based on *F*^2^ are statistically about twice as large as those based on *F*, and *R*- factors based on ALL data will be even larger.

### Fractional atomic coordinates and isotropic or equivalent isotropic displacement parameters (Å^2^)

**Table:**

	*x*	*y*	*z*	*U*_iso_*/*U*_eq_	
Cl1A	0.46391 (11)	0.7593 (6)	0.94032 (9)	0.0625 (6)	
Cl2A	0.31181 (11)	0.9079 (6)	0.65527 (9)	0.0630 (6)	
N1A	0.5405 (3)	0.4243 (18)	0.7726 (3)	0.0560 (15)	
C2A	0.5808 (4)	0.369 (2)	0.8223 (4)	0.0544 (18)	
H2AA	0.6274	0.2581	0.8219	0.065*	
C3A	0.5587 (4)	0.467 (2)	0.8763 (4)	0.0560 (18)	
H3AA	0.5892	0.4193	0.9108	0.067*	
C4A	0.4924 (4)	0.6317 (19)	0.8765 (3)	0.0501 (16)	
C5A	0.3766 (4)	0.8694 (19)	0.8220 (4)	0.0544 (18)	
H5AA	0.3579	0.9425	0.8560	0.065*	
C6A	0.3371 (4)	0.9282 (19)	0.7711 (4)	0.0517 (17)	
H6AA	0.2906	1.0420	0.7694	0.062*	
C7A	0.3646 (4)	0.822 (2)	0.7200 (4)	0.0541 (18)	
C8A	0.4305 (4)	0.652 (2)	0.7206 (4)	0.0531 (17)	
H8AA	0.4475	0.5771	0.6860	0.064*	
C9A	0.4735 (4)	0.5904 (19)	0.7737 (4)	0.0506 (16)	
C10A	0.4461 (4)	0.6984 (18)	0.8250 (3)	0.0498 (16)	
Cl1B	0.50197 (10)	0.9725 (5)	0.58983 (9)	0.0589 (6)	
Cl2B	0.80689 (11)	0.2033 (5)	0.48184 (10)	0.0625 (6)	
N1B	0.7313 (3)	0.6479 (18)	0.6687 (3)	0.0559 (16)	
C2B	0.6835 (4)	0.802 (2)	0.6981 (4)	0.0569 (18)	
H2BA	0.6984	0.8463	0.7373	0.068*	
C3B	0.6115 (4)	0.908 (2)	0.6755 (4)	0.0527 (17)	
H3BA	0.5797	1.0195	0.6991	0.063*	
C4B	0.5891 (4)	0.848 (2)	0.6205 (4)	0.0528 (17)	
C5B	0.6209 (4)	0.615 (2)	0.5258 (4)	0.0559 (19)	
H5BA	0.5730	0.6680	0.5077	0.067*	
C6B	0.6720 (4)	0.472 (2)	0.4941 (4)	0.0539 (17)	
H6BA	0.6609	0.4309	0.4544	0.065*	
C7B	0.7417 (4)	0.388 (2)	0.5232 (4)	0.0565 (19)	
C8B	0.7602 (4)	0.439 (2)	0.5800 (4)	0.0527 (17)	
H8BA	0.8074	0.3700	0.5978	0.063*	
C9B	0.7092 (4)	0.5951 (19)	0.6121 (3)	0.0483 (16)	
C10B	0.6378 (4)	0.6840 (19)	0.5843 (4)	0.0526 (17)	

### Atomic displacement parameters (Å^2^)

**Table:**

	*U*^11^	*U*^22^	*U*^33^	*U*^12^	*U*^13^	*U*^23^
Cl1A	0.0557 (11)	0.0571 (11)	0.0757 (13)	0.0000 (8)	0.0113 (9)	−0.0010 (9)
Cl2A	0.0476 (10)	0.0611 (11)	0.0788 (13)	0.0022 (8)	0.0012 (8)	0.0029 (9)
N1A	0.036 (3)	0.050 (3)	0.082 (4)	−0.002 (3)	0.010 (3)	0.001 (3)
C2A	0.041 (4)	0.049 (4)	0.076 (5)	0.001 (3)	0.018 (3)	0.002 (3)
C3A	0.043 (4)	0.044 (4)	0.080 (5)	−0.004 (3)	0.001 (3)	0.008 (3)
C4A	0.045 (4)	0.044 (3)	0.063 (4)	−0.005 (3)	0.013 (3)	−0.005 (3)
C5A	0.041 (4)	0.038 (3)	0.086 (5)	−0.002 (3)	0.014 (3)	−0.004 (3)
C6A	0.035 (3)	0.044 (3)	0.077 (5)	−0.001 (3)	0.012 (3)	−0.001 (3)
C7A	0.039 (4)	0.045 (4)	0.078 (5)	−0.005 (3)	0.003 (3)	0.004 (3)
C8A	0.045 (4)	0.043 (4)	0.072 (5)	−0.002 (3)	0.013 (3)	−0.007 (3)
C9A	0.036 (3)	0.039 (3)	0.077 (5)	−0.004 (3)	0.011 (3)	−0.001 (3)
C10A	0.043 (4)	0.038 (3)	0.069 (4)	−0.005 (3)	0.007 (3)	−0.002 (3)
Cl1B	0.0374 (9)	0.0560 (10)	0.0837 (13)	0.0058 (7)	0.0087 (8)	0.0054 (9)
Cl2B	0.0474 (10)	0.0558 (10)	0.0875 (14)	0.0036 (8)	0.0205 (8)	−0.0008 (9)
N1B	0.038 (3)	0.049 (3)	0.080 (4)	−0.002 (3)	0.005 (3)	0.006 (3)
C2B	0.040 (4)	0.047 (4)	0.083 (5)	−0.002 (3)	0.005 (3)	0.006 (4)
C3B	0.035 (3)	0.052 (4)	0.072 (5)	−0.004 (3)	0.011 (3)	0.004 (3)
C4B	0.041 (4)	0.044 (4)	0.074 (5)	−0.005 (3)	0.012 (3)	0.011 (3)
C5B	0.041 (4)	0.050 (4)	0.075 (5)	−0.008 (3)	−0.003 (3)	0.011 (3)
C6B	0.038 (4)	0.058 (4)	0.068 (5)	−0.004 (3)	0.016 (3)	0.001 (3)
C7B	0.031 (3)	0.048 (4)	0.093 (6)	−0.003 (3)	0.018 (3)	0.005 (4)
C8B	0.034 (3)	0.044 (4)	0.081 (5)	−0.001 (3)	0.008 (3)	0.002 (3)
C9B	0.034 (3)	0.042 (3)	0.069 (4)	−0.002 (3)	0.008 (3)	0.006 (3)
C10B	0.034 (3)	0.040 (3)	0.084 (5)	−0.002 (3)	0.008 (3)	0.006 (3)

### Geometric parameters (Å, º)

**Table:**

Cl1A—C4A	1.720 (8)	Cl1B—C4B	1.734 (8)
Cl2A—C7A	1.742 (8)	Cl2B—C7B	1.769 (8)
N1A—C2A	1.328 (11)	N1B—C2B	1.313 (11)
N1A—C9A	1.379 (10)	N1B—C9B	1.368 (10)
C2A—C3A	1.432 (12)	C2B—C3B	1.418 (11)
C2A—H2AA	0.9500	C2B—H2BA	0.9500
C3A—C4A	1.362 (11)	C3B—C4B	1.338 (12)
C3A—H3AA	0.9500	C3B—H3BA	0.9500
C4A—C10A	1.422 (11)	C4B—C10B	1.443 (11)
C5A—C6A	1.348 (12)	C5B—C6B	1.373 (12)
C5A—C10A	1.420 (11)	C5B—C10B	1.407 (12)
C5A—H5AA	0.9500	C5B—H5BA	0.9500
C6A—C7A	1.417 (12)	C6B—C7B	1.410 (11)
C6A—H6AA	0.9500	C6B—H6BA	0.9500
C7A—C8A	1.364 (11)	C7B—C8B	1.359 (12)
C8A—C9A	1.421 (11)	C8B—C9B	1.400 (11)
C8A—H8AA	0.9500	C8B—H8BA	0.9500
C9A—C10A	1.422 (11)	C9B—C10B	1.429 (10)
			
C2A—N1A—C9A	117.2 (8)	C2B—N1B—C9B	116.3 (7)
N1A—C2A—C3A	124.2 (7)	N1B—C2B—C3B	124.9 (8)
N1A—C2A—H2AA	117.9	N1B—C2B—H2BA	117.6
C3A—C2A—H2AA	117.9	C3B—C2B—H2BA	117.6
C4A—C3A—C2A	117.7 (7)	C4B—C3B—C2B	118.8 (8)
C4A—C3A—H3AA	121.1	C4B—C3B—H3BA	120.6
C2A—C3A—H3AA	121.1	C2B—C3B—H3BA	120.6
C3A—C4A—C10A	121.2 (8)	C3B—C4B—C10B	120.7 (7)
C3A—C4A—Cl1A	119.5 (7)	C3B—C4B—Cl1B	121.4 (6)
C10A—C4A—Cl1A	119.4 (6)	C10B—C4B—Cl1B	117.9 (6)
C6A—C5A—C10A	120.2 (8)	C6B—C5B—C10B	121.7 (7)
C6A—C5A—H5AA	119.9	C6B—C5B—H5BA	119.1
C10A—C5A—H5AA	119.9	C10B—C5B—H5BA	119.1
C5A—C6A—C7A	120.5 (7)	C5B—C6B—C7B	117.0 (8)
C5A—C6A—H6AA	119.8	C5B—C6B—H6BA	121.5
C7A—C6A—H6AA	119.8	C7B—C6B—H6BA	121.5
C8A—C7A—C6A	121.7 (8)	C8B—C7B—C6B	123.7 (7)
C8A—C7A—Cl2A	119.7 (7)	C8B—C7B—Cl2B	119.8 (6)
C6A—C7A—Cl2A	118.6 (6)	C6B—C7B—Cl2B	116.5 (7)
C7A—C8A—C9A	118.9 (8)	C7B—C8B—C9B	119.4 (7)
C7A—C8A—H8AA	120.6	C7B—C8B—H8BA	120.3
C9A—C8A—H8AA	120.6	C9B—C8B—H8BA	120.3
N1A—C9A—C8A	117.3 (8)	N1B—C9B—C8B	117.0 (7)
N1A—C9A—C10A	123.3 (7)	N1B—C9B—C10B	124.4 (7)
C8A—C9A—C10A	119.5 (7)	C8B—C9B—C10B	118.7 (7)
C4A—C10A—C5A	124.2 (8)	C5B—C10B—C9B	119.3 (7)
C4A—C10A—C9A	116.4 (7)	C5B—C10B—C4B	125.8 (7)
C5A—C10A—C9A	119.3 (7)	C9B—C10B—C4B	114.9 (7)
			
C9A—N1A—C2A—C3A	0.9 (12)	C9B—N1B—C2B—C3B	1.5 (12)
N1A—C2A—C3A—C4A	−1.0 (12)	N1B—C2B—C3B—C4B	0.3 (12)
C2A—C3A—C4A—C10A	1.1 (11)	C2B—C3B—C4B—C10B	−1.2 (11)
C2A—C3A—C4A—Cl1A	−179.2 (6)	C2B—C3B—C4B—Cl1B	−179.5 (6)
C10A—C5A—C6A—C7A	−0.2 (11)	C10B—C5B—C6B—C7B	1.7 (12)
C5A—C6A—C7A—C8A	1.1 (12)	C5B—C6B—C7B—C8B	0.4 (12)
C5A—C6A—C7A—Cl2A	−179.4 (6)	C5B—C6B—C7B—Cl2B	179.9 (6)
C6A—C7A—C8A—C9A	−1.7 (11)	C6B—C7B—C8B—C9B	−2.4 (12)
Cl2A—C7A—C8A—C9A	178.8 (6)	Cl2B—C7B—C8B—C9B	178.1 (6)
C2A—N1A—C9A—C8A	179.4 (7)	C2B—N1B—C9B—C8B	178.4 (7)
C2A—N1A—C9A—C10A	−0.9 (11)	C2B—N1B—C9B—C10B	−2.5 (11)
C7A—C8A—C9A—N1A	−178.8 (7)	C7B—C8B—C9B—N1B	−178.5 (7)
C7A—C8A—C9A—C10A	1.4 (11)	C7B—C8B—C9B—C10B	2.3 (11)
C3A—C4A—C10A—C5A	−179.7 (7)	C6B—C5B—C10B—C9B	−1.7 (11)
Cl1A—C4A—C10A—C5A	0.5 (10)	C6B—C5B—C10B—C4B	177.0 (7)
C3A—C4A—C10A—C9A	−1.1 (11)	N1B—C9B—C10B—C5B	−179.5 (7)
Cl1A—C4A—C10A—C9A	179.2 (5)	C8B—C9B—C10B—C5B	−0.3 (11)
C6A—C5A—C10A—C4A	178.6 (7)	N1B—C9B—C10B—C4B	1.7 (11)
C6A—C5A—C10A—C9A	0.0 (11)	C8B—C9B—C10B—C4B	−179.2 (7)
N1A—C9A—C10A—C4A	1.0 (10)	C3B—C4B—C10B—C5B	−178.5 (7)
C8A—C9A—C10A—C4A	−179.3 (7)	Cl1B—C4B—C10B—C5B	−0.1 (10)
N1A—C9A—C10A—C5A	179.7 (7)	C3B—C4B—C10B—C9B	0.3 (10)
C8A—C9A—C10A—C5A	−0.6 (10)	Cl1B—C4B—C10B—C9B	178.6 (5)
